# Prevalence of Severe Visual Disability Among Preterm Children With Retinopathy of Prematurity and Association With Adherence to Best Practice Guidelines

**DOI:** 10.1001/jamanetworkopen.2018.6801

**Published:** 2019-01-04

**Authors:** Mikael Norman, Ann Hellström, Boubou Hallberg, Agneta Wallin, Pelle Gustafson, Kristina Tornqvist, Stellan Håkansson, Gerd Holmström

**Affiliations:** 1 Division of Pediatrics, Department of Clinical Science, Intervention and Technology, Karolinska Institutet, Stockholm, Sweden; 2 Department of Neonatal Medicine, Karolinska University Hospital, Stockholm, Sweden; 3 Swedish Neonatal Quality Registry, Umeå, Sweden; 4 Department of Ophthalmology, Institute of Neuroscience and Physiology, The Sahlgrenska Academy, Gothenburg University, Gothenburg, Sweden; 5 Department of Pediatric Ophthalmology and Strabismus, St Erik Eye Hospital, Stockholm, Sweden; 6 The Swedish National Patient Insurance, Stockholm, Sweden; 7 Department of Clinical Sciences, Ophthalmology, Lund University, Lund, Sweden; 8 Department of Clinical Science/Pediatrics, Umeå University, Umeå, Sweden; 9 Department of Neuroscience/Ophthalmology, Uppsala University, Uppsala, Sweden

## Abstract

**Importance:**

Retinopathy of prematurity (ROP) can cause severe visual disability even in high-resource settings. A better understanding of the prevalence and processes leading to ROP-induced severe visual impairment may help health care professionals design preventive measures.

**Objectives:**

To determine the prevalence of severe visual disability among children born preterm in Sweden, evaluate adherence to best practice, and determine the health system’s structural capacity.

**Design, Setting, and Participants:**

Population-based, nationwide cohort study of 1 310 227 children born between January 1, 2004, and December 31, 2015, in Sweden, of whom 17 588 (1.3%) were born very preterm (<32 weeks of gestation). Children born preterm with a verified diagnosis of severe visual disability had their medical records reviewed for evaluation of ROP screening, diagnosis, and treatment. In addition, a questionnaire on structural capacity was sent to all ophthalmology departments.

**Exposures:**

Stages 4 and 5 ROP.

**Main Outcomes and Measures:**

The primary outcome was prevalence of severe visual disability (visual acuity ≤20/200 for both eyes) associated with ROP stages 4 and 5. Secondary outcomes included adherence to national ROP guidelines using a predefined protocol with 15 key performance indicators for screening, diagnosis, and treatment; assessment of whether visual disability was deemed avoidable; and examination of structural capacity, including information on equipment and facilities, staffing, and patients.

**Results:**

Seventeen children (10 boys; mean [range] birth weight, 756 [454-1900] g; mean [range] gestational age, 25 [22-33] weeks) became severely visually disabled because of ROP, corresponding to a prevalence of 1 in 1000 very preterm infants (<32 weeks of gestational age) and 1 in 77 000 for all live births. Severe visual impairment was considered potentially avoidable in 11 of 17 affected children (65%) owing to untimely or no screening, missed diagnosis, or untimely and suboptimal treatment. Large variations in infrastructure (facilities, guidelines, staffing, and annual patient numbers) were also identified as potential contributors to these findings.

**Conclusions and Relevance:**

Retinopathy of prematurity still causes severe visual disability in Sweden, resulting in 1 affected infant per 1000 very preterm births. In most of these infants, noncompliance with best practice was identified, indicating that a significant proportion could have been avoided.

## Key Points

**Question:**

What is the prevalence of visually disabling retinopathy of prematurity in Sweden and how is it associated with quality of care?

**Findings:**

In this population-based cohort study of 1 310 227 infants born alive in Sweden in 2004 to 2015, 1 in 77 000 or 1 in 1000 very preterm infants (<32 weeks of gestational age) became severely visually disabled by retinopathy of prematurity. In 11 of 17 infants (65%), disability was considered avoidable because of nonadherence to best practice, as well as flaws in structural capacity.

**Meaning:**

Severe visual impairment associated with retinopathy of prematurity is preventable in most cases.

## Teaser

This cohort study investigates the prevalence of severe visual disability due to retinopathy of prematurity (ROP) among children in Sweden and examines the association between prevalence of disability and adherence to best practice guidelines for treatment of ROP.

## Introduction

Retinopathy of prematurity (ROP) is a major cause of lifelong visual disability among children born preterm, particularly in resource-poor settings. Globally, an estimated 20 000 infants become severely visually disabled from ROP each year, and ROP has become an increasing cause of blindness in China, Southeast and South Asia, Latin America, and parts of Eastern Europe.^1^ Given effective tools for screening, diagnosis, and treatment, severe disability due to ROP should be possible to avoid in most infants. However, disabling ROP still occurs even in high-resource settings.^2,3^ Therefore, a better understanding of the structures and processes leading to disabling ROP is needed.

In regions defined as developed by the World Health Organization Millennium Development Goals, an estimated 1 200 000 babies are born preterm each year.^4^ Among the 150 000 believed to be at risk for developing ROP, 1700 are estimated to become severely visually disabled, giving a prevalence of disabling ROP in high-resource settings of 1 in 90 to 1 in 100 very preterm (<32 weeks of gestation) births.^1^ However, there is only limited information on disabling ROP from population-based studies. Although the prevalence of severe ROP may vary in relation to case mix and to varying approaches to oxygen saturation targets, monitoring, and nutrition policies,^5,6,7^ less is known about the progression to disabling ROP. In a previous Swedish study of extremely preterm infants, 20% of those with an indication for ROP treatment were treated in an untimely manner, indicating that nonadherence to best practice may play a role.^8^

We know of no previous studies on severely disabling ROP combining population-based epidemiology to establish the true prevalence with analysis of adherence to best practice and underlying structural capacity. In 2008, national guidelines for screening, diagnosis, and treatment of ROP were issued based on population studies,^9,10,11^ including the Swedish National Register for Retinopathy of Prematurity (SWEDROP).^12^ Using these and other sources of registry and database information, the aim of this study was to determine the prevalence of severely disabling ROP in a population of more than 1 000 000 children, determine adherence to best practice in infancy, and decide to what extent severe visual disability could be considered avoidable. We also aimed to capture information from all ophthalmology departments in Sweden regarding their present structural capacity.

## Methods

Reporting of this study followed the Standards for Quality Improvement Reporting Excellence (SQUIRE) 2.0 reporting guideline. Ethical approval for this study was obtained from the Regional Ethics Review Board in Stockholm, Sweden, on August 10, 2016. Informed consent was not obtained and a waiver of such consent was granted by the Regional Ethics Review Board.

### Population

This nationwide population-based cohort study comprised all live-born infants, including those delivered preterm (<37 weeks of gestation) or very preterm (<32 weeks of gestation) in Sweden during a 12-year study period between January 1, 2004, and December 31, 2015. Using data from several sources, we identified all preterm infants with stage 4 or 5 ROP who developed severe visual disability (defined as visual acuity ≤20/200 for both eyes).^13^ Severe visual disability from causes other than stage 4 or 5 ROP (such as cerebral visual impairment, optic atrophy, refractive errors, strabismus and congenital infections, chromosomal disorder, or malformations) was an exclusion criterion (n = 0 in our search output). Children with visual disability residing in Sweden but born outside the country were also excluded (n = 1).

### Data Sources

Open-access statistics provided by the National Board of Health and Welfare were used to assess the total number of preterm births in the study period. We used SWEDROP^12^ and the Swedish Visual Disability Register^14^ to identify preterm infants with stage 4 or 5 ROP (n = 25). The SWEDROP registry was initiated in 2006 but did not have full national coverage (approximately 95%) until 2008. We therefore used the Swedish Neonatal Quality Registry and the Extremely Preterm Infants in Sweden Study (EXPRESS) database^8,15,16^ to identify preterm infants with stage 4 or 5 ROP born in 2004 to 2007 (n = 30). In addition, we extracted all files of complaints within the Swedish National Patient Insurance database with *International Classification of Diseases, Tenth Revision,* codes for ROP or blindness (n = 10). This registry and database search provided 65 eligible infants.

### Medical Record Review

Personal identification numbers retrieved from the registers and databases were used to access medical records at hospital databases and county archives. Records from both the neonatal units and the ophthalmology departments were scrutinized according a predefined protocol by 5 of us (M.N., A.H., B.H., A.W., and G.H.). First, misclassifications of ROP, gestational age, or date of birth were checked for. Second, we reviewed the medical records for present visual status and included only those with severe visual disability (n = 17). Third, we applied the exclusion criteria (n = 1 born outside Sweden). The flowchart for inclusion can be seen in the Figure. Among children with visual disability, we could not get access to medical records in 3 cases. These participants were evaluated using available registry and database information.

**Figure.**
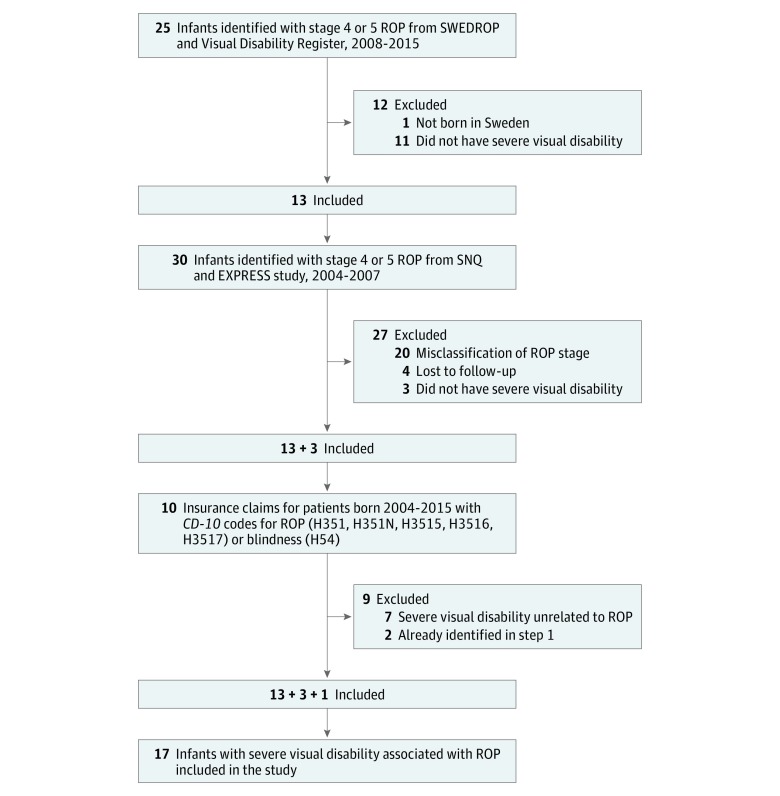
Flowchart of Participants EXPRESS indicates Extremely Preterm Infants in Sweden Study; *ICD-10, International Classification of Diseases, Tenth Revision*; ROP, retinopathy of prematurity; SNQ, Swedish Neonatal Quality Registry; SWEDROP, Swedish National Register for Retinopathy of Prematurity.

Using national^17^ and international^18,19,20,21^ guidelines, we identified 15 key performance indicators: 5 related to documentation, 5 related to ROP screening, and 5 related to ROP diagnosis and treatment (Table 1). Based on these indicators, we established a predefined protocol of best practice in management of ROP and applied this protocol to our medical record review. Although not outlined in our guideline, we considered fewer than 1500 laser photocoagulation effects per eye as an indication of suboptimal density to prevent disease progression at first treatment.^19^

**Table 1.** Adherence to National Guidelines and Best Practice in the Neonatal Period Among 17 Children Who Developed Severe Visual Disability Due to ROP

Key Performance Indicator^a^	No./Total No. (%)
Documentation in neonatal unit records	
Documentation of ROP stage only	11/14 (79)
Documentation included zone or type	3/14 (21)
ROP screenings documented in neonatal record	6/13 (46)
ROP treatment documented in neonatal record	6/13 (46)
If transfer to other hospitals, ROP screenings and treatment clearly documented	4/11 (36)
Screening	
For infants with GA at birth ≥27 wk, first screening examination at 5 wk postnatal age	1/2 (50)
If GA at birth <27 wk, first screen no later than 31 wk postmenstrual age	11/14 (79)
Screening exams repeated >2 weeks apart	3/13 (23)
Postponed screening exams	5/14 (36)
If transferred to other hospital, timely screening without interruption	4/11 (36)
Diagnosis and treatment	
Missed indication for treatment	6/17 (35)
Timely treatment (within 72 h of diagnosis)	7/15 (47)
Aggressive ROP treated within 48 h	5/12 (42)
First treatment with <1500 laser photocoagulation applications	6/12 (50)
Postoperative retinal examination within 5-7 d	10/15 (67)
Additional information on treatment	
Second laser treatment	12/14 (86)
Anti–vascular endothelial growth factor administered	2/17 (12)
Vitrectomy or encircling band	10/17 (59)

Abbreviations: GA, gestational age; ROP, retinopathy of prematurity.

^a^ Some data missing or nonapplicable (3 children evaluated using available registry and database information but medical records were unavailable).

The World Health Organization defines patient safety as “the absence of preventable harm to a patient during the process of health care and reduction of risk of unnecessary harm associated with health care to an acceptable minimum. An acceptable minimum refers to the collective notions of given current knowledge, resources available, and the context in which care was delivered.”^22^ Based on compliance with best practice and using the classification of the National Board of Health and Welfare in Sweden,^23^ we characterized cases of disabling ROP as unavoidable, possibly unavoidable, possibly avoidable (estimated probability ≥51%) or avoidable (estimated probability ≥75%). The categorization of avoidability was performed in the format of a roundtable workshop with 5 of us—2 senior neonatologists (M.N. and B.H.) and 3 experienced ophthalmologists (A.H., A.W., and G.H.)—scrutinizing the medical records. All decisions were made in consensus.

### Questionnaire to Ophthalmology Departments

To evaluate current health care systems’ capacity, a questionnaire was sent to all ophthalmology departments (n = 35) in Sweden in 2017, including 2 reminders. The questionnaire contained 26 questions relating to infrastructure, facilities, equipment, staffing, and education, as well as number of patients and ROP examinations and treatments.

### Statistical Analysis

Data are presented as number and proportions (percentages), mean (SD), or median (range) values. Calculations were performed using Microsoft Excel 2013 software.

## Results

### Prevalence of Severely Disabling ROP in Sweden

Seventeen children (10 boys; mean [range] birth weight, 756 [454-1900] g; mean [range] gestational age, 25 [22-33] weeks) became severely visually disabled because of ROP, corresponding to a prevalence of 1 in 1000 very preterm infants (<32 weeks of gestational age) and 1 in 77 000 of all live births. In total, there were 1 310 227 live born infants in Sweden in 2004 to 2015. Of these, 79 488 (6.1%) were born before 37 weeks of gestation and 17 588 (1.3%) before 32 weeks of gestation. Among children with visual disability, 7 were born in 2004 to 2009 (the first 6 years of the study period), and 10 were born in 2010 to 2015, of whom 8 were born in 2013 to 2015.

### Perinatal Characteristics

Perinatal characteristics and selected information on ROP stage and treatment in children with severe disability due to ROP are presented in Table 2. Most perinatal characteristics were similar to those reported in a previous population-based cohort of infants born before 27 weeks of gestation in Sweden,^15^ ie, the infants who became visually disabled did not differ from the general extremely preterm population except that almost all (94%) had bronchopulmonary dysplasia. One child with visual disability was born at 33 weeks’ gestation with severe lung hypoplasia secondary to a large diaphragmatic hernia, illustrating that ROP requiring treatment may also occur in more mature infants with exposure to high fraction of inspired oxygen for a prolonged period (in this case >3 months).

**Table 2.** Perinatal Characteristics of 17 Children in Sweden With Severe Visual Disability due to ROP, 2004 to 2015

Maternal Characteristics	No. (%)
Maternal age, mean (SD) [range], y	30.5 (6.8) [20-41]
Primiparous mother	8 (47)
Pregnancy and delivery	
Diabetes in pregnancy	0
Multiple pregnancy	1 (6)
Preeclampsia	2 (12)
Any antenatal corticosteroids	16 (94)
Delivery at level III unit	12 (71)
Cesarean delivery	7 (41)
Duration of gestation, mean (SD) [range], wk	24.8 (2.7) [22-33]
Infant characteristics	
Boys	10 (59)
Birth weight, mean (SD) [range], g	756 (341) [454-1900]
Small for gestational age	3 (18)
Neonatal morbidity	
Intraventricular hemorrhage grade 3-4 or cystic periventricular leukomalacia	2 (12)
Patent ductus arteriosus, treated	14 (83)
Patent ductus arteriosus surgery	9 (53)
Septicemia	10 (59)
Necrotizing enterocolitis	0
Bronchopulmonary dysplasia	16 (94)
Hospital transfers	
≥2 Transfers between hospitals	11 (65)
ROP stages and treatment	
ROP stage 5 both eyes	9 (53)
ROP stage 5 one eye	2 (12)
ROP stage 4 one or two eyes	6 (35)
Laser applications for first treatment, No.	
Mean (SD)	1340 (547)
Median (range)	1324 (203-2048)
Laser applications for second treatment, No.	
Mean (SD)	617 (422)
Median (range)	430 (172-1151)

Abbreviation: ROP, retinopathy of prematurity.

### Adherence to Best Practice

Auditing the medical records of the 17 children who developed severe visual disability, we found several flaws in documentation, screening, and treatment. Poor documentation in neonatal records, postponing screening examinations, and delayed screening after transfer between hospitals were of particular concern. Untimely and suboptimal treatment also occurred, and follow-up after first treatment was delayed in 1 of 3 infants (Table 1).

### Avoidable Visual Disability

We identified 6 possibly avoidable and 5 avoidable injuries of severe visual impairment due to ROP. In 1 infant, both screening and treatment failed, meaning that severe visual disability in 11 of 17 children (65%) most likely could have been avoided had best practice care been provided. Examples of avoidable visual disability included situations in which screening was never performed or had been interrupted for up to 6 weeks, retinal detachment was not recognized in spite of weekly examinations for a month after detachment had occurred, and treatment was delayed or clearly insufficient (Table 3). Laser coagulation was used as first-line treatment in 16 of 17 infants (all but 1 infant who was 33 weeks gestational age and was never screened, in whom retinal detachment was discovered at 4.5 months of postnatal age). Additional treatment included encircling band (n = 6), vitrectomy (n = 3), and anti–vascular endothelial growth factor (n = 2).

**Table 3.** Nonadherence to Guidelines and Avoidable Patient Injury

Type of Noncompliance to Key Performance Indicator	Infants, No.		
	Total	Visual Disability Possibly Avoidable	Visual Disability Avoidable
Untimely screening (never done, started too late, or postponed several times)	5	0	3^a^
Missed diagnosis, staging, or grading	2	0	2
Treatment untimely or suboptimal	10	6	1^a^

^a^ One infant with both untimely screening and suboptimal treatment.

### Financial Compensation

During the study period, 3 of 11 families (27%) affected by possibly avoidable or avoidable visual disability due to ROP applied for financial compensation from the Swedish National Patient Insurance. Two families who had applied for financial compensation were subsequently granted it.

### Capacity of the Health Care System

In total, 25 of 35 ophthalmology departments responded to our questionnaire, including all 6 university centers in Sweden. Several of the nonresponders were smaller hospitals. One responder from a small hospital reported that they had no very preterm patients in their hospital, and responses from this hospital were therefore excluded from further analysis.

Table 4 summarizes structure and capacity for ROP screening, diagnosis, and treatment in Sweden. Of particular note, only one-fifth of the Swedish units reported regular use of a retinal camera for ROP screening and only 3 hospitals transferred retinal photographs to university centers. Twelve percent of departments had fewer than 10 annual patients and 21% of ophthalmologists performed fewer than 25 annual ROP screening examinations. Documentation and guidelines also varied. Treatment appeared to be centralized, demanding swift collaboration between hospitals and administrations to manage time limits.

**Table 4.** Survey on Structure for ROP Screening, Diagnosis, and Treatment in 24 Swedish Ophthalmology Departments

Infrastructure and Capacity	No. (%)
**Patients**	
Very preterm infants screened in hospital annually, No.	
<10	3 (12)
10-50	15 (62)
>50	6 (25)
**Equipment**	
Retinal camera	
Available in the facility	14 (58)
Used for screening	5 (21)
Photographs transferred to center of excellence	3 (12)
Documentation	
Results of ROP screenings documented in both ophthalmology and neonatal medical record	7 (29)
Guidelines	
Written guidelines for screening, diagnosis, and treatment of ROP	23 (94)
Written standard for notifying ROP surgeon	9 (38)
Written standard for reporting ROP status when patient is transferred to other hospital	5 (21)
**Staffing**	
Ophthalmologists for ROP screenings in department, No.	
1-2	7 (29)
3-4	14 (58)
≥5	3 (12)
ROP screening examinations performed per ophthalmologist annually, No.	
<25	5 (21)
25-50	12 (50)
>50	7 (29)
**Treatment Facilities**	
Treatment of ROP performed	
At the facility	6 (25)
At regional center	10 (42)
Outside region	8 (33)
Laser or anti–vascular endothelial growth factor treatments performed at facility annually, No.	
0	18 (75)
1-4	1 (4)
5-10	3 (12)
>10	2 (8)

Abbreviation: ROP, retinopathy of prematurity.

## Discussion

This national population-based registry study revealed 4 clinically important findings: first, ROP causing severe visual disability still occurs in Sweden with a prevalence 1 of 1000 children being born very preterm. Second, in 2 of 3 children with visual impairment due to ROP, the visual disability was considered to have been avoidable owing to suboptimal clinical performance characterized by screening and treatments that were offered too little too late. Third, although most likely entitled to financial compensation, only a minority (27%) of families affected by avoidable or possibly avoidable visual injury in their children submitted requests for compensation to the Swedish Patient Insurance. Fourth, our survey disclosed large variations in national infrastructure regarding screening, diagnosis, and treatment for ROP, which may have contributed to substandard care and adverse outcomes.

The prevalence of severe visual disability secondary to ROP was one-tenth of that recently estimated from meta-analyses pooling information from high-income countries.^1^ This discrepancy was surprising given the comparatively high survival rate among extremely preterm infants, the high incidence of ROP, and high prevalence of later visual problems found in this group of Swedish children.^2,7^ Other European population-based studies of children surviving extremely preterm birth have reported similar results as in our study,^24,25^ suggesting that the prevalence of disabling ROP in high-resource settings may be overestimated in global surveys.^1^

Despite national guidelines that have been continuously improved and modified national guidelines since 1993,^9,10,11,17^ there were 17 children with end-stage ROP causing severe visual impairment, with no decline in prevalence over time. With few exceptions, the children with visual disability exhibited well-known risk factors for severe ROP, such as birth after extremely short gestation (22-26 weeks) and neonatal exposure to supplemental oxygen for prolonged periods, underlining a need for meticulous screening and proactive treatment.^8^ Only 2 children had neonatal brain injury (intraventricular hemorrhage grade 3 or cystic periventricular leukomalacia), and in both cases we considered the severe visual disability to be mainly associated with ROP, as these infants had retinal detachment (ROP stage 4-5). As shown in EXPRESS,^2,3^ the total prevalence of severe visual disability among extremely preterm survivors would be approximately twice that reported herein, if adding severe visual disability secondary to comorbidities of prematurity (such as severe neonatal brain injury) to ROP.

The structured medical record review performed by 3 experienced pediatric ophthalmologists and 2 neonatologists revealed deviations from national recommendations regarding both ROP screening and treatment procedures. Successful ROP management requires adherence to best practice, including timely start and frequency of the screening examinations. This requires a close collaboration between the neonatologist and the ophthalmologist and is particularly important to consider when transferring the infant to other hospitals. In children with severe disability due to ROP, we found that ROP screenings and treatments had been poorly documented in neonatal records, and that only 1 in 3 neonatal records contained specific information on ROP when the patients had been transferred between hospitals. Delayed screening and lack of screening were associated with severe visual disability in 3 children.

The most common deviation from best practice that was associated with potentially avoidable visual disability in our study was untimely or suboptimal ROP treatment. Once criteria for treatment have been fulfilled, treatment must be undertaken in a timely manner, ie, within 72 hours. This requires not only excellent collaboration between ophthalmologists and neonatologists, but also with anesthetists, as laser treatment is generally performed under general anesthesia. In addition, only 1 of 4 departments reported that ROP treatment could be provided in the facility, a challenging structural emergency for transport organizations, hospitals, and administrations, as well as for families. Furthermore, and very importantly, treatment has to be adequate, with an appropriately placed and sufficiently large number of laser applications, covering the avascular, peripheral retinal area. Finally, follow-up after treatment is crucial to identify infants with so-called skip areas in need of further laser treatment or treatment with other modalities (anti–vascular endothelial growth factor injections or, in case of retinal detachment, vitreoretinal surgery).^26,27^

Our study revealed large variations in infrastructure for ROP screening and treatment, including number of patients, equipment, and staffing. Professional evaluation of the fundus of preterm infants requires a critical mass of yearly examinations and treatments. Although standards have not been defined by numbers so far, we find fewer than 10 annual patients (12% of departments) and fewer than 25 annual ROP screenings per ophthalmologist (21% of departments) worryingly low, as the competence of the screening and treating ophthalmologist is crucial to avoid unfavorable visual outcome, something that is underlined in the British ROP guidelines.^20^ Documentation of the findings with the wide-angle retinal camera is also helpful and enables digital communication with diagnostic experts as well as with those performing treatment. Surprisingly, only one-fifth of the Swedish units reported regular use of a retinal camera for ROP screening and only 3 hospitals transfer retinal photos to university hospitals where treatment is performed.

In addition to problems posed by a shortage of experienced experts in ROP examinations, infants at risk are geographically dispersed once they have been back-transferred from regional hospitals to step-down neonatal units closer to the families’ homes. A more widespread use of retinal cameras and telemedicine could therefore be a way to improve the quality of ROP screening in Sweden. Telemedicine has been reported to be a safe, reliable, and cost-effective complement to the efforts of ophthalmologists in Canada^28^ and is currently under evaluation in a larger context.^29,30^

In Sweden, patients who experience an injury while in the health care system may be entitled to financial compensation under the Patient Injury Act. Approximately 16 000 injuries are reported to the Patient Injury Insurance each year, and 40% of them are compensated. Avoidable severe disability originating in the neonatal period is one of the most highly compensated conditions. Only 3 of 11 families (27%) with avoidable or potentially avoidable visual disability applied for financial compensation, whereas the remaining 8 families never applied to the Patient Injury Insurance. This could reflect lack of awareness among families and professionals despite the fact that health care professionals are obliged to inform patients and parents about this public insurance. It may also reflect a reluctance among physicians to acknowledge severe neonatal complications as patient injuries.

In 2008 to 2015, 4 of 1000 preterm infants born in Sweden at less than 31 weeks of gestational age have been reported to be affected by stage 4 or 5 ROP.^7^ Adding our findings to these data indicate that a majority with stage 4 or 5 ROP do not become severely visually disabled. Many of these children have acceptable visual function at follow-up, at least in 1 eye. In addition, most infants with ROP who are managed suboptimally are likely to avoid severe visual disability.

The strengths of this study include the large population base and the use of personal identification numbers to link information from several registers and databases and validate this information against medical records. We included all preterm infants born in Sweden over more than a decade and collected information on several neonatal diagnoses, enabling us to present truly population-based prevalence of severe visual disability due to ROP as distinct from severe visual disability caused by other comorbidities. Including not only ROP stage but also final visual ability, we critically evaluated practice using a predefined protocol based on national guidelines in all children with the most unfavorable outcome, ie, severe visual impairment. Combining registry data and medical record reviews with a contemporary department survey, we were able to identify several potential areas of improvement in clinical practice as well as in the structure of the health system.

### Limitations

Our study also has some limitations. It is retrospective in design and therefore the registers as well as medical records may have lacked some information. We had no access to photographs to verify adequacy of laser treatment, and some centers may have used lasers with a larger spot size, requiring fewer spots. In addition, the number of delivered spots may have been higher than those that were actually focused and became spots. Therefore, our limit of fewer than 1500 spots as an indication of suboptimal density to prevent disease progression at first treatment could be insufficient, contributing to our more cautious conclusion that flaws in treatment were possibly avoidable. Our structured medical record reviews were limited to worst-case scenarios and say little if anything about clinical performance regarding ROP in general. The survey on infrastructure and capacity was self-reported, which could introduce bias. In Sweden, there is no regular ophthalmological follow-up of all children born very preterm, only for those who have been treated for ROP or had major neonatal brain injury.

## Conclusions

Despite national guidelines for ROP screening and treatment, children born preterm still become severely visually impaired in Sweden. Although the prevalence is low in an international comparison, severe visual disability could have been avoided in more than half of the children had there been better adherence to best practice, a better structural capacity, and higher competence among ophthalmologists and neonatologists. We request that screening and treatment for ROP be prioritized by departments and hospital administrations, including continuous education and training of the ophthalmologists screening and treating these infants. To avoid lifelong visual disability in preterm infants, equal care within different parts of a country should be sought, and centralization of the most difficult cases and treatment may be a way forward.
